# Can changes in malaria transmission intensity explain prolonged protection and contribute to high protective efficacy of intermittent preventive treatment for malaria in infants?

**DOI:** 10.1186/1475-2875-7-54

**Published:** 2008-04-03

**Authors:** Roly D Gosling, Azra C Ghani, Jaqueline L Deen, Lorenz von Seidlein, Brian M Greenwood, Daniel Chandramohan

**Affiliations:** 1 Department of Infectious and Tropical Disease, London School of Hygiene and Tropical Medicine, London, UK; 2 MRC Centre for Outbreak Analysis & Modeling, Department of Infectious Disease Epidemiology, Imperial College London, London, UK; 3 International Vaccine Institute, Seoul, South Korea

## Abstract

**Background:**

Intermittent preventive (or presumptive) treatment of infants (IPTi), the administration of a curative anti-malarial dose to infants whether or not they are known to be infected, is being considered as a new strategy for malaria control. Five of the six trials using sulphadoxine-pyrimethamine (SP) for IPTi showed protective efficacies (PEs) against clinical malaria ranging from 20.1 – 33.3% whilst one, the Ifakara study, showed a protective efficacy of 58.6%.

**Materials and methods:**

The possible mechanisms that could explain the differences in the reported PE of IPTi were examined by comparing output from a mathematical model to data from the six published IPTi trials.

**Results:**

Under stable transmission, the PE of IPTi predicted by the model was comparable with the observed PEs in all but the Ifakara study (ratio of the mean predicted PE to that observed was 1.02, range 0.39 – 1.59). When a reduction in the incidence of infection during the study was included in the model, the predicted PE of IPTi increased and extended into the second year of life, as observed in the Ifakara study.

**Conclusion:**

A decrease in malaria transmission during the study period may explain part of the difference in observed PEs of IPTi between sites and the extended period of protection into the second year of life observed in the Ifakara study. This finding of continued benefit of interventions in settings of decreasing transmission may explain why rebound of clinical malaria was absent in the large scale trials of insecticide-treated bed nets.

## Background

Intermittent preventive treatment of infants (IPTi) is the administration of a curative anti-malarial dose to infants, whether or not they are known to be infected, at specified times to prevent malaria [1]. IPTi delivered through the EPI programme was first shown to successfully prevent malaria in infants in 2001 [2]. Three doses of sulphadoxine-pyrimethamine (SP) given to Tanzanian infants living in an area of perennial transmission at the time of vaccination with DPT2, DPT3 and measles vaccines reduced the incidence of clinical malaria and anaemia during the first year of life by 59% and 50% respectively. Furthermore, protection against clinical episodes of malaria persisted into the second year of life [3]. In contrast, in northern Ghana, where malaria transmission is intense and highly seasonal, SP-IPTi gave only 25% protection against clinical malaria and 35% protection against hospital admissions with anaemia during the first year of life and no protection during the second year [4]. A similar level of protection against clinical malaria during the first year of life was seen in Mozambique but no protection against anaemia was detected in this study [5]. Further trials of SP-IPTi conducted in areas of Ghana [6,7] and Gabon [8] with differing epidemiological patterns of malaria have given similar results to those observed in Ghana and Mozambique. The results from the first study in Tanzania therefore appear at odds with those from the later studies.

A number of explanations for the differences in protective efficacy (PE) of IPTi against clinical malaria between sites has been suggested including the intensity of transmission and consequent malaria incidence, the pattern of antimalarial resistance, the administration of iron and the use of additional control measures, specifically insecticide-treated nets (ITN) [9]. This paper, using data from the six SP-IPTi randomized placebo-controlled trials reported so far, explored the association between resistance to SP, ITN coverage and malaria transmission intensity in each study site. The observed PE of IPTi against clinical malaria is examined using a mathematical model which mimics the acquisition and loss of parasites to predict the PE expected in the six trial settings.

## Methods

### Data

Data are from IPTi trials conducted in Manhiça (Mozambique), Lambarene (Gabon), Ifakara (Tanzania) and Navrongo, Kumasi and Tamale (Ghana). Detailed descriptions of the study population, methodology and outcome in each study included in this analysis have been published elsewhere [2-8]. A summary of the study designs and their epidemiological background is shown in Table 1. The model output was compared to data derived from the IPTi Consortium's Statistical Working Group (SWG) Report of September 2007. The SWG used common definitions for time at risk and for an episode of clinical malaria across all six studies. For time at risk a child treated for clinical malaria was censured for 21 days in order to prevent double counting of cases and to allow for any prophylactic effect of the antimalarial. A case of clinical malaria was defined as measured fever or history of fever with any parasitaemia of *P. falciparum *(definition of duration of history of fever differed between studies: for Ifakara and Manhica studies it was 24 hours and for remaining studies it was 48 hours). In this paper, all references to the PE of IPTi refers to the PE against episodes of clinical malaria up to 12 months of age based on incidence rates of multiple episodes of clinical malaria, not time to first or only episodes.

**Table 1:** Study characteristics of SP-IPTi efficacy trials

**Study parameter**	**Schellenberg *et al***. [2, 3]	**Chandramohan *et al***. [4]	**Macete *et al ***[5]	**Kobbe *et al***. [6]	**Mockenhaupt *et al***. [7]	**Grobusch *et al ***[8]
Trial, country	Ifakara, Tanzania	Navrongo, Ghana	Manhica, Mozambique	Kumasi, Ghana	Tamale, Ghana	Lambaréné, Gabon
Recruitment year(s)	1999–2000	2000–2002	2002–2004	2003–2005	2003	2004–2005
EIR/year	29	418	38	400	NA	50
Transmission	Perennial moderate	Highly seasonal high	Perennial with seasonal peaks moderate	Perennial high	Perennial with seasonal peaks high	Perennial with seasonal peaks low-moderate
*In vivo *SP resistance by day 14%	31 (1999–2000) [10]	22 (2004) [11]	21 (2001) [12]	NA	14 (2002) [14]	21 (2004) [13]
Use of bed nets, % placebo/SP treated (untreated)	67/68	17/19	0/0 (14/15)	20/20 estimate (39/38)	<1%	5/5 (80/80)
Iron supplementation	Yes	Yes	None	None	None	None
Ages at dosing, months	2, 3, 9 (at time of DPT2, DPT3 & measles)	3, 4, 9, 12 (at time of DPT2, DPT3 & measles + extra at 12 months)	3, 4, 9 (at time of DPT2, DPT3 & measles)	3, 9, 15 (at time of DPT3 & measles + extra at 15 months)	3, 9, 15 (at time of DPT3 & measles + extra at 15 months)	3, 9, 15 (at time of DPT3 & measles + extra at 15 months)
No. of children enrolled, placebo/active	351/350 = 701	1,242/1,243 = 2,485	755/748 = 1,503	535/535 = 1,070	600/600 = 1,200	595/594 = 1,189
Study design	Individual randomization	Cluster randomization	Individual randomization	Individual randomization	Individual randomization	Individual randomization

The relationship between the observed PE of IPTi and the following potential determinants of PE were explored: resistance to SP; estimated ITN coverage (% of the study population reporting use of ITN); and malaria transmission intensity (mean incidence of malaria per child per year in the placebo group). Day-14 parasitological and clinical failure rates were used to define resistance because five out of the six IPTi trials had published this information within two years of conducting the IPTi trial [10-14]. One site in Ghana, Kumasi, did not have data on day 14 parasitological and clinical failure of SP and therefore the estimate from Tamale, relatively close geographically, was used.

### Mathematical model

**Figure 1:**
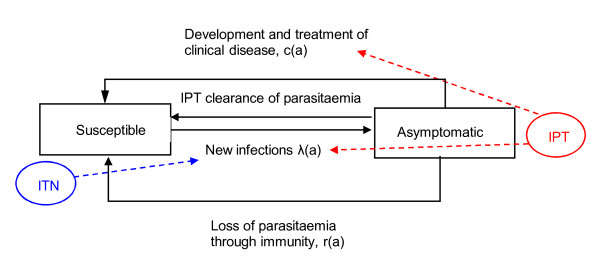
The asymptomatic parasite pool model.

An age-structured model (Figure 1) was developed to represent the acquisition of malaria infection and clinical disease and the development of immunity in the study cohort of infants between the ages of two and 24 months. The modelling exercise only examines the specific cohort as studied in the trials, thus age and calendar times are equivalent and the model output is expressed in terms of the age of the children. At any point in time children can be in one of two states – uninfected and susceptible to new infection S(a) or infected with parasites which can remain asymptomatic or can become symptomatic, A(a). It is assumed that the rate of acquisition of new infections is determined by the force of infection in the study area, *λ*(a), which may vary through time (and hence by age). Once infected and in the asymptomatic state, children return to the susceptible state through one of three routes. First, they may become a clinical case and receive an effective treatment. It is assumed that in clinical trial settings, every case of malaria detected was adequately treated and parasites cleared. Secondly, they may receive antimalarial treatment for asymptomatic parasitaemia eg IPTi. Finally, they may remain asymptomatic and recover naturally. Symptomatic cases of malaria that are not detected by surveillance systems will remain in the asymptomatic state in the model until they die of severe disease or their immune response clears parasites. The model does not incorporate children leaving the asymptomatic pool by death, assuming this will be a very small number because most of the cases would be detected in time to receive effective treatment in a trial setting.

Ignoring mortality from other causes and migration, the model without interventions can be expressed by the following equation:

$$\frac{dA(a)}{da}=\lambda (a)(N-A(a))-(c(a)+r(a))A(a)$$

where N is the fixed population size, r(a) is the age-dependent rate of natural clearance of parasitaemia and c(a) is the age-dependent rate of development of clinical disease which is then treated.

In endemic areas the risk of developing clinical disease decreases with exposure to infection but rates of parasitaemia remain almost constant in early childhood. The model incorporates functions that mimic the development of immunity so that as children age the rate at which they develop clinical disease decreases and the rate at which they clear parasites increases. For simplicity, the model assumes that both immunity functions are linearly dependent on the expected number of malaria infections at age a:

$$E[I(a)]=\frac{1}{N}\int_{0}^{a}\lambda (a')S(a')da'$$

The rate of development of clinical disease is given by the logistic function

$$c(a)=\phi \frac{\alpha_{1}+1}{\alpha_{1}+e^{\alpha_{2}E[I(a)]}}$$

where *ϕ *is the rate of development of clinical disease in the absence of immunity and *α*_1 _and *α*_2 _are parameters which determine the number of infections after which full immunity to clinical disease occurs. The rate of natural clearance of parasites is assumed to be linear within the range of interest and hence is given by

*r*(*a*) = min(*ωE *[*I*(*a*)], 1))

where 1/*ω *is the mean number of infections after which full parasite immunity is obtained and it was assumed that at full immunity parasites are cleared after a mean of one day.

A generic maternal protective function which acts to reduce the force of infection following birth was incorporated. Maternal protection is complicated and multifaceted [15], incorporating both biological immunity as well as behavioural factors that limit exposure. Given the paucity of data with which to determine an appropriate function, the following factors were used, which act on the force of infection up to six months of age: 0.05, 0.15, 0.4 and 0.8 at age 2, 3, 4 and 5 months of age respectively, which represent a gradual loss of immunity.

The model was numerically evaluated as difference equations in 1-month time-steps using Excel.

### Incorporation of IPT and ITN

To compare the model results to the trial data the two interventions were incorporated. Firstly, use of ITNs is included by reducing the force of infection in the group assigned to ITNs by a factor *θ*:

$$\frac{dA(a)}{da}=(1-\theta )\lambda (a)(N-A(a))-(c(a)+r(a))A(a)$$

The model only examines the personal protection gained from an ITN and does not examine any other effects, such as effects on transmission.

IPT use across all age-groups modelled (two to 24 months) is assumed to act in three ways, clearing parasites in a proportion (1-*σ*) of the population, ie the treatment effect, prophylaxes against new infection (factor *σ *reducing the force of infection) and reducing the rate of development of clinical disease by the same factor. As the model is defined in monthly time-steps, the parameter *σ *can be interpreted as the PCR uncorrected day 28 Adequate Clinical and Parasitological Response (ACPR) which measures the proportion that will clear parasitaemia and be protected against new infection 28 days post treatment. For simplicity, it was assumed that the ACPR acts with equal efficacy to clear parasites, to protect against new infection and prevent development of disease.

$$\frac{dA(a)}{da}=(1-\sigma )\lambda (a)(N-A(a))-((1-\sigma )c(a)+r(a))A(a)-\sigma A(a)$$

Equation (6) is applied to the model only for months when IPTi doses are given. For months in those children who used ITNs and receive IPT equations (5) and (6) are combined.

For the modelling exercise, the coverage of ITNs (reported ownership) in each trial site was used because the use of ITN at the individual level was not available for all trials. The expected incidence of clinical disease in each trial arm (placebo and IPT) is therefore calculated as a weighted combination of the model predictions with and without ITNs. The protective efficacy of IPT predicted by the model is calculated as 1-relative risk = 1-clinical incidence in IPTi group/clinical incidence in placebo group.

### Model parameters

It is assumed that that on average after 5 infections of malaria an individual is totally protected against clinical disease and that after 50 infections an individual can clear parasites rapidly. A sensitivity analysis for these parameters can be found later in this paper. Parameters that reproduce these patterns are given in Table 2. The force of infection, *λ*(a) was assumed to be constant for the baseline scenarios. At a later stage the modelling exercise used scenarios in which the force of infection was decreased in early childhood due to maternal protective factors and separately allowed to increase or decrease linearly as the children aged reflecting changes in transmission over time. The force of infection was initially estimated directly as the mean incidence of clinical malaria in the placebo group. This approximation was based on the observation that the age-specific incidence of clinical malaria is highest in infants, the IPTi target age group and that the age group with the highest incidence should have the lowest immunity. The observed data from the Navrongo study (the only full dataset available to the study team) was compared with the model estimates of clinical disease, which were found to be half of that observed in the study. Thus, the mean incidence of clinical malaria in the placebo group was multiplied by a factor of two to estimate the force of infection. Entomological Inoculation Rates (EIR) were not used as a measure of transmission in the model for two reasons: EIRs measured concurrently with the IPTi trials were not available for most sites and in the studies with EIRs there was no common methodology. Secondly, these large cohort studies had enrolled children from a large geographical area over several years (two to four years). Thus a single measure of EIR would not suffice to represent the whole study area or the whole study period.

**Table 2:** Summary of Model Parameters and Symbols

Parameter Description	Parameter Symbol	Value
***Definitions for equations***		
Susceptible population at age (a)	S(a)	N/A
Asymptomatic population at age (a)	A(a)	N/A
Age-dependent rate of natural clearance of parasitaemia at age (a)	r(a)	N/A
Age-dependent rate of development of clinical disease which is then treated at age (a)	c(a)	N/A
Protective Efficacy	PE	N/A
***Fixed parameters***		
Rate of development of clinical disease in the absence of immunity	*φ*	0.9
Mean number of infections after which full immunity to clinical disease occurs	*μ*	5
Parameters which determine the number of infections after which full immunity to clinical disease occurs	*α*_1 _and *α*_2_	1
Mean number of infections after which full parasite immunity occurs	*ω*	50
Protection offered by ITN use	*θ*	0.5
***Variable parameters between sites***		
Force of infection	*λ*(a)	See table 3
Drug action reducing the force of infection	*σ*	See table 3

The proportion of infected children becoming symptomatic and treated in the absence of immunity was assumed to be 90% in one month. This was derived from a study of asymptomatic parasitaemia in 6–59 month old children in a moderate malaria setting in Kampala, Uganda [16]; in this population 50% of children with asymptomatic parasitaemia developed clinical malaria after 30 days. As the Ugandan study was undertaken in partially immune children we assumed a higher rate of development of disease. Clinical malaria cases are assumed to recover within a month post treatment, twice the average terminal half-life of the antimalarials used for treatment and rejoin the susceptible population. Deaths and migrations were not included in the model.

For those children receiving IPT, it is assumed that treatment, prophylaxis, and prevention of developing clinical disease effects of SP will be equally affected by the PCR uncorrected day 28 ACPR of SP. Day 28 PCR uncorrected ACPR is a measure of both the treatment and prophylactic effect combined (it includes both recrudescence's and re-infections) and is more likely to represent the effects of the drugs when used for prevention as opposed to treatment. The sensitivity analysis for how changes in ACPR affect PE is shown in the in the results section. Briefly, as drug resistance increases PE declines. The day 28 ACPR was only available for 2 sites, the sites with the highest and lowest resistances at day 14, namely Ifakara [10] and Tamale [14] respectively. The extrapolation from day 14 to 28 efficacy for the 3 studies [11-13] without day 28 ACPR is the mid point between these two studies.

For those children using ITNs, we assume that the protective efficacy of an ITN is 0.5 [17]. The model parameters are summarised in Table 2.

### Sensitivity analysis

A sensitivity analysis of how ACPR, ITN coverage and immunity functions affects predicted PE was carried out.

## Results

### Association between IPTi protective efficacy and various factors

**Figure 2:**
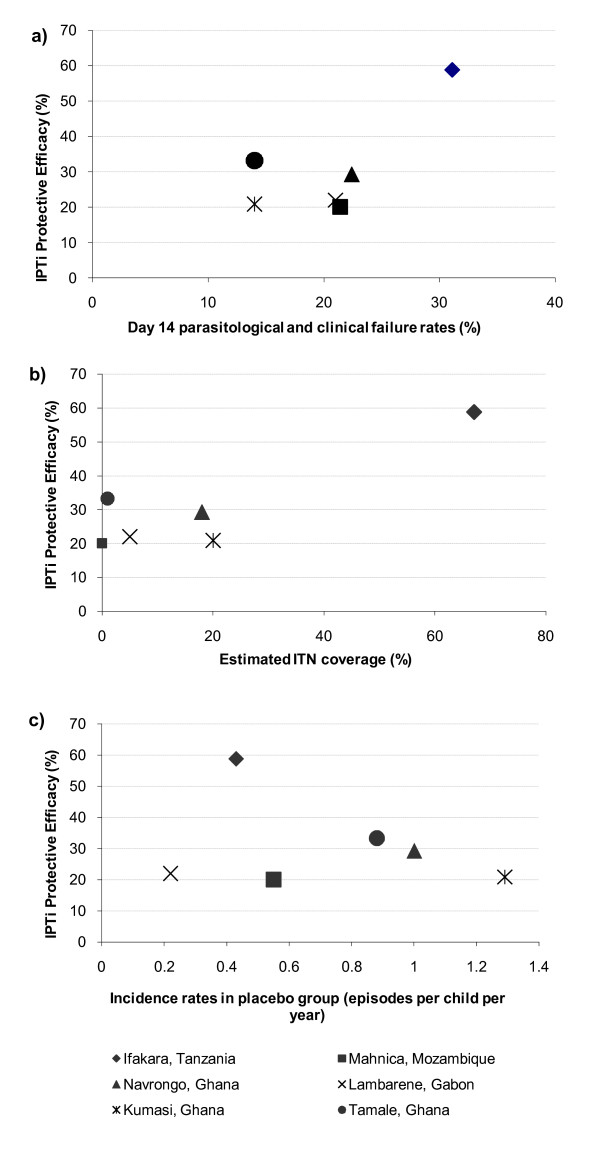
Protective efficacy of IPTi at 12 months of age compared to estimated resistance to SP at Day 14, ITN coverage and incidence of malaria in placebo groups.

Figure 2 shows the relationship between PE of IPTi and resistance to SP, estimated ITN coverage, and malaria transmission intensity in each study site. The Ifakara study site had the highest IPTi PE (59%) despite having the highest resistance to SP (31% day-14 parasitological and clinical failure rate). This site also had the highest ITN coverage (67%). Resistance to SP was 14 – 22% and ITN coverage was 0 – 20% in the other five sites.

### Protective efficacy of IPTi in stable transmission settings

**Figure 3:**
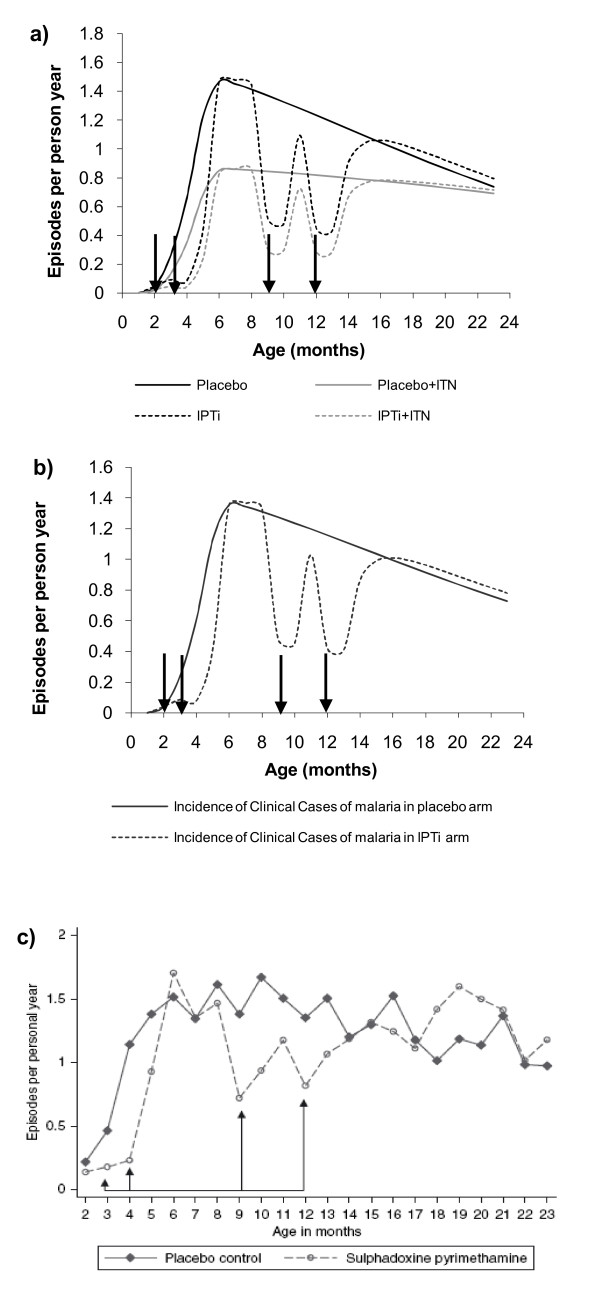
**Asymptomatic pool model prediction of monthly clinical cases per child year at risk from the Navrongo, Ghana IPTi study (A) by intervention group and ITN use by age with stable transmission (B) the prediction weighted by ITN coverage and (C) the actual incidence by age in the placebo and IPTi groups from the Navrongo study [18] (by kind permission of Tropical Medicine and International Health, Blackwell Publishing).** Arrows indicate time of IPTi dosing.

The model predicted a similar pattern across the six trials, with a transient decline in incidence among the groups receiving IPTi (with and without ITN coverage) as well as a generally lower incidence among groups with ITNs (with and without IPTi). Using the Navrongo study as an example (IPTi doses given at 3, 4, 9 and 12 months of age), Figure 3 shows the models prediction of monthly incidence of clinical disease cases in groups with and without ITNs (Figure 3A), the combined model weighted by ITN coverage (Figure 3B) and observed data from the Navrongo [18] study for comparison (Figure 3C). In those trial settings with higher incidence the model generates a small increase in the numbers of cases shortly after each IPTi dose due to delayed acquisition of immunity which continues into the second year of life (the rebound effect).

The predicted PE of IPTi in the six sites with the observed data are shown in Table 3. The mean ratio between model to actual PE was 1.02 (range 0.39 – 1.59) with the predicted PE lying within the 95% confidence interval from the trial data in all but the Ifakara study. The main differences between the Ifakara and the other studies were the high ITN coverage and the higher resistance to SP. There seems no obvious explanation to why IPTi should be more effective with higher drug resistance. However, high ITN coverage may have an effect on transmission. Thus the effect of changing transmission on the PE predicted by the model is further explored.

**Table 3:** Modelled and actual protective efficacy to 12 months of age in each IPTi trials

	**Input**					**Output**		
**Study Site**	**Age of SP IPTi administration (months)**	**Mean incidence in placebo group (episodes per person year) (*λ*(a)/2)**	**ITN coverage (%)**	Estimated*** cross sectional prevalence parasitaemia at start of study (%)	**Estimated Day 28 ACPR for SP (*σ *× 100)**	**Model estimate of PE (%)**	**Actual PE of IPTi (% 95% CI)**	**Ratio model:study**

Ifakara	2,3 and 9	0.54	67	4.5	60	23.0	58.8 (40.8–71.3)	0.39
Navrongo	2,4,9 and 12	1	18	8.3	65*	31.9	29.3 (17.7–39.5)	1.09
Mahnica	3,4 and 9	0.71	0	5.9	65*	32.0	20.1 (2.1–34.9)	1.59
Kumasi	3,9 and 15	1.27	20	10.6	69**	25.9	20.9 (8.9–31.3)	1.24
Tamale	3,9 and 15	0.93	1	7.8	69	24.9	33.3 (20.7–43.8)	0.75
Lamberene	3,9 and 15	0.16	5	1.3	65*	23.7	22.0 (-25.4–51.5)	1.08

* Estimated from Day 14 ACPR, ** Estimated from Tamale data.

*** Starting Cross sectional parasite prevalence (asymptomatic infected children) estimated from mean monthly incidence in placebo group Incidence and PE figures from IPTi Consortium Statistical Working Group Report September 2007 (Aponte JJ et al. In preparation)

### Protective efficacy of IPTi in changing transmission settings

**Figure 4:**
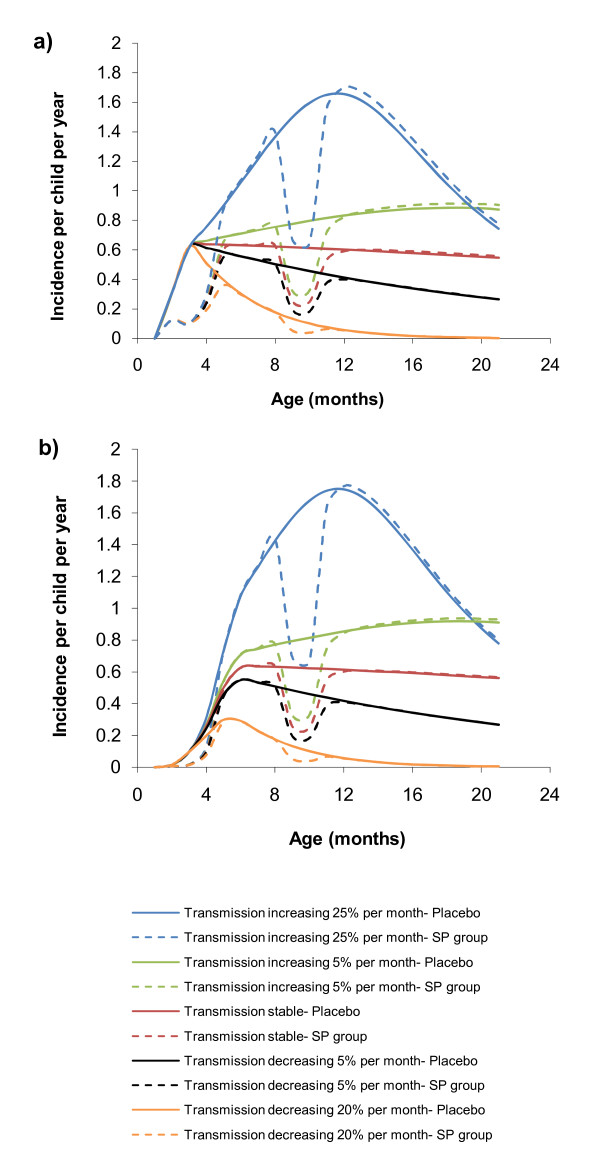
Model predictions of Ifakara Tanzania IPTi study without (A) and including (B) maternal immunity function with different changes in transmission.

Table 4 shows the change in both PE and the effect of delayed immunity (rebound effect) predicted by the model for Ifakara under four scenarios with changing transmission during the study period with both maternal immunity function removed and included – (a) increasing at a rate of 25% per month, (b) increasing at a rate of 5% per month, (c) stable, (d) a decline of 5% per month and (e) a decline of 25% per month. In the scenarios of changing transmission, PE is dominated when the effect of IPTi is most efficacious, ie when transmission is highest. In the output of the model without the maternal immunity function (Figure 4A) the overall PE measured by the trial is dominated by its early efficacy with the first two doses of IPTi. Thus, because most of the efficacy is predicted to occur during the months in which doses are given (at 2, 3 and 9 months of age in this trial), the measured overall PE is high. Conversely, if transmission is increasing then the overall measure of PE will be dominated by what is happening during the later months when doses are no longer being given and hence a lower overall protective efficacy will be estimated. When including the maternal immunity function (Figure 4B) the 3^rd ^dose of IPTi given at nine months of age has the dominant effect on the PE. Changes in transmission affect the extent to which rebound or prolonged protection are observed in the months following the IPTi doses independently of maternal immunity. If overall transmission is being reduced, this in turn reduces the probability of infection once the direct protection afforded by IPTi is removed and hence reduces the potential for the rebound effect to be observed and increases the probability of observing prolonged protection.

**Table 4:** Change in Protective Efficacy and rebound effect with changes in transmission

**Transmission**	**Transmission effect per month (%)**	**Predicted protective efficacy over first 12 months (%)**	**Protection until**	**Rebound effect**
***No maternal immunity***				
Fast increase	1.25 (25)	27.1	12 months	Yes
Increasing	1.05 (5)	29.2	12 months	Yes
Stable	1 (0)	30.6	13 months	Yes
Slow decline	0.95 (5)	32.2	13 months	No
Fast decline	0.75 (25)	42.8	> 24 months	No
***Maternal immunity included***				
Fast increase	1.25 (25)	22.7	12 months	Yes
Increasing	1.05 (5)	22.1	13 months	Yes
Stable	1 (0)	23.0	14 months	Yes
Slow decline	0.95 (5)	22.7	14 months	No
Fast decline	0.75 (25)	23.1	16 months	No

### Sensitivity analysis

The results of the sensitivity analysis of ACPR are shown in Table 5. PE increases when the ACPR is high (i.e. there is little resistance) as the IPT effect is greatest under this scenario and visa versa. Varying ITN coverage from 0–100% had little effect on predicted PEs of the trials (range of variation of PE from baseline (results shown in Table 3): 0–0.6%). Increasing the mean number of infections to become immune against clinical disease from 5 to 10 reduced predicted PE but the magnitude was small (range of variation of PE from baseline: 0.3–0.8%). Varying the effect of number of infections to get anti-parasite immunity also had little effect on predicted PE (range of number attacks required to get anti-parasite immunity: 20–100, range of variation of PE from baseline: 0–0.3%). The maternal immunity function greatly affected predicted PE. Table 6 shows the effect on PE when (a) no immunity is predicted, (b) the models fixed non-parametric form is used (baseline) and (c) a function of maternal immunity against severe disease published elsewhere [19]. Without maternal immunity PE is enhanced.

**Table 5:** Sensitivity analysis of effects of ACPR on models predictions of PE

Study Site	Observed PE (%, 95% CI)	Model PE (%)		
		Baseline	ACPR increased to 100%	ACPR reduced to 40%

Ifakara	58.8 (40.8–71.3)	23.0	37.5	15.5
Manhica	20.1 (2.1–34.9)	32.0	47.4	20.2
Navrongo	29.3 (17.7–39.5)	31.9	47.3	20.1
Lamebarene	22.0 (-25.4–51.5)	23.6	36.4	14.5
Kumasi	20.9 (8.9–31.3)	25.9	36.9	15.3
Tamale	33.3 (20.7–43.8)	24.9	36.5	14.3

**Table 6:** Sensitivity analysis of maternal immunity function on models predictions of PE with (a) no maternal immunity function, (b) with fixed non-parametric function used in the paper (Baseline) and (c) function based on maternal immunity to severe disease.

Study Site	Observed PE (%, 95% CI)	Model PE (%)		
		(a) No immunity	(b) Baseline	(c) Alternative based on immunity to severe disease

Ifakara	58.8 (40.8–71.3)	32.4	23.0	26.4
Manhica	20.1 (2.1–34.9)	38.6	32.0	34.0
Navrongo	29.3 (17.7–39.5)	38.5	31.9	34.1
Lamebarene	22.0 (-25.4–51.5)	28.2	23.6	26.0
Kumasi	20.9 (8.9–31.3)	38.0	25.9	30.3
Tamale	33.3 (20.7–43.8)	29.7	24.9	27.4

## Discussion

The high PE of IPTi found in the Ifakara study and a similar preventive trial using amodiaquine in north-eastern Tanzania [20] triggered a series of IPT trials in other African study sites to investigate this potentially promising method of malaria control. Subsequent published trials showed a much lower efficacy of IPTi than was observed in Ifakara [2]. To explain these differences in efficacy between sites some observers have focussed on the differences in drug resistance to SP between the sites. However, this explanation does not appear plausible because the site with the highest PE had the highest SP resistance (Figure 2). In response to this observation, it has been suggested that there may be an immunisation effect of SP, the "Leaky Drug" theory [3,21]. The hypothesis is that a partially effective drug allows for low level and persisting parasitaemia and thus allowing prolonged stimulation of the immune system resulting in the extended period of protection as seen in the Ifakara site. This model-based analysis provides an alternative explanation, namely that the exceptionally high ITN coverage in Ifakara decreased transmission and boosted the observed PE of IPTi. High ITN coverage was recognised as a potential explanation of differences in PE between the Manhica and Ifakara studies [9]. Ifakara District is known to have experienced a 10 fold reduction in transmission around the study period (for example, the EIR in 1995 was recorded as 300 and by 2001 had fallen to 29). Although the EIR estimates came from different places within the district there was a reported change in the epidemiology of clinical disease during this time period [22]. In addition many other studies have shown the mass effect on transmission of high ITN coverage [17]. The model suggests that changing the transmission intensity affects both the PE and the length of protection and thus gives a plausible explanation for the difference in results between study sites. Another modelling exercise focussing on the mechanism of IPTi (Ross A., manuscript in preparation) has confirmed this finding. No clear decrease was seen in the mean incidence of clinical malaria in the placebo arm of the Ifakara study from the published data from the first [2] to the second [3] year, going from 0.43 to 0.42 episodes per person year at risk. The model predicts that over the first year of the study transmission must fall by at least 22% per month to be within the 95% confidence limits of the PE observed. Whilst this seems unlikely, the pattern of transmission faced by the cohort may have changed within the observation period and affected the observed PE. To test the hypothesis derived from this model the data will need to be examined by looking at monthly incidence in each group by age in the Ifakara study.

The model shows that PE mainly depends on the level of malaria transmission during the few months which IPTi doses are administered and the length of follow up and transmission intensity when IPTi is not given. To maximise PE IPTi should be given during high malaria transmission and follow up should be short when malaria transmission is low. Supportive evidence for this is demonstrated in the extended analysis of the Navrongo study [18] and an IPT seasonal study where antimalarials were given in Senegal, West Africa during the malaria seasons with a short follow up of 13 weeks [23]. In this study efficacy against clinical malaria was 86%.

This model also provides a coherent explanation as to why no rebound effect would be observed in situations of decreasing transmission, such as Ifakara or Kenya [24,25]. The delay in acquisition of immunity caused by very successful interventions such as continuous chemoprophylaxis in infants are followed by increases in cases following cessation of the intervention, the rebound effect [26-28]. In this situation of chemoprophylaxis in a single age group there is no effect on transmission. However, in the large ITN trials where no rebound was seen, the mass effect of the ITNs in reducing vectorial capacity led to a decrease in transmission [17]. The model predicts that in the presence of decreasing transmission rebound parasitaemia can disappear. Thus, although the population is immunologically more susceptible to infection with malaria, it is less exposed and so cases of malaria infection reduce. In contrast, when an intervention that reduces exposure and hence immunity to malaria takes place in a site with stable malaria transmission or one in which transmission is increasing a rebound effect would be evident. If IPT was spread across all age groups, ie as a form of mass drug administration or universal IPT (IPTu) reducing the asymptomatic pool (A(a) in the model) in the whole population and not a small age group, an effect on transmission may be seen.

ITNs exert a steady personal protection to the individual sleeping under the net of approximately 50% [17] as long as the insecticide remains active. IPTi offers intermittent protection which varies with the efficacy of the drug only at times when it is administered. Therefore it follows that protection with an ITN should be the primary intervention with IPTi as an additional strategy. The model found the largest difference in incidence of clinical malaria between placebo groups without ITN compared to ITN plus IPTi. This observation suggests that combining interventions must be a priority. ITN coverage has little influence on the predicted PE by the model. This is because the model defines the protection of an ITN to act to reduce the force of infection to the proportion of those using ITNs and then calculates the overall PE weighted by this coverage. The model assumes an additive effect of IPTi and ITNs and not synergy.

As with all theoretical studies, the model has some limitations. The model is dependent on some key assumptions regarding the effect of exposure on immunity. First, it is assumed that full clinical immunity was obtained after five infections. This figure was derived from past estimates for severe malaria [19] but clearly requires further data for verification. Increasing the number of infections required to become immune to developing clinical disease would result in a smaller rebound effect and a smaller decrease in transmission would eliminate the rebound effect. No clear evidence for rebound has been seen in the trials [29], thus the number of clinical attacks leading to immunity is likely to be more than five. Similarly, the threshold for achieving parasite immunity was arbitrarily set at 50 infections. However, as the model only considers malaria in the first 2 years of life, children are unlikely to reach the five attacks needed to become immune to clinical disease (mean number of expected attacks in Kumasi, the highest transmission setting, was 2.7 at 24 months of age) and even less likely to reach the 50 attacks to give full antiparasite immunity, the results are less sensitive to these choices of immune function. Whilst the choice of immunity functions determines the extent of the rebound effect predicted by the model, it does not impact greatly on the protective efficacies predicted by the model. In contrast, the assumptions made regarding maternal protection do impact on the predicted protective efficacy (Tables 4 and 6, Figure 4A and 4B). The effect of maternal protection is likely to vary by site and be influenced by levels of transmission experienced by the mother during the transplacental passage of humoral immunity and behavioural factors. Further data are required to refine this function. Differences in calculating time at risk following treatment also bias the model to detect a higher PE. In the analysis used to produce the PEs in the studies, a child was censored for 21 days after each case of malaria yet the model uses one month time steps and so cases of malaria are censored seven days longer, reducing the time at risk denominator. The model studies very few variables and only those directly affecting malaria. The differences of the PEs in the studies could be related to factors so far remaining unstudied such as HIV prevalence, socio-economic status, timing and dose of IPTi, heterogeneity of malaria transmission or placental infection.

What does this study mean for IPTi? This model demonstrates that during a decline in malaria transmission, which Africa is currently experiencing, IPTi can be highly effective and safe. Combining IPTi with ITNs results in greater protection for an individual, further more, if high levels of coverage of ITNs can be attained with a resultant decrease in transmission, then there appears to be synergy between the interventions. However, if transmission subsequently increases a reduction in the efficacy of IPTi (as currently measured by comparing incidence rates of malaria over a long period) can be expected. In stable conditions, PE does not seem to be greatly affected by levels of transmission, however the higher the level of transmission the more likely a rebound effect is to be seen. The rebound effect is equivalent to delaying of clinical cases of malaria to an older age which may be beneficial as older children appear to develop less severe illness than infants [28]. Indeed, these observations will apply to all types of interventions during times of changing transmission intensity. Currently there is a reduction of transmission across sub-Saharan Africa [30], thus exposure and immunity will be reduced leaving the possibility for outbreaks of malaria disease should transmission increase again. Drug resistance does play a role in IPTi efficacy and this should continue to be monitored. However, changes in transmission are likely to have a greater effect on IPTi protective efficacy in the trials that have taken place with the levels of drug resistance studied.

## Competing interests

The authors declare no conflict of interest. RG, DC and BG are members of the IPTi Consortium. The views expressed in the paper are those of the authors and not of the IPTi Consortium.

## Authors' contributions

RG conceived the idea and developed the model and wrote the paper. AG developed the concept, refined the model and wrote the paper. JD developed the model and wrote the paper. LVS develop the concept and wrote the paper. BG reviewed the early manuscript and wrote the paper. DC developed the concept and model and wrote the paper. All authors read and approved the final version.
